# Carglumic acid as a treatment for persistent hyperammonemia in carnitine-acylcarnitine translocase deficiency: A case study

**DOI:** 10.1016/j.ymgmr.2025.101199

**Published:** 2025-02-07

**Authors:** Hanım Babazade, Tanyel Zubarioglu, Esma Uygur, Mehmet Şerif Cansever, Ertuğrul Kiykim, Çiğdem Aktuğlu Zeybek

**Affiliations:** a İstanbul University-Cerrahpaşa, Cerrahpaşa Medical Faculty, Department of Pediatrics, Division of Nutrition and Metabolism, İstanbul, Turkey; b İstanbul University-Cerrahpaşa, Cerrahpaşa Medical Faculty, Research Laboratory of Metabolism, İstanbul, Turkey; c İstanbul University-Cerrahpaşa, The Vocational School of Health Services, Department of Medical Documentation and Techniques, Division of Medical Laboratory Techniques, İstanbul,Turkey

**Keywords:** Fatty acid oxidation disorders, Hyperammonemia, Carglumic acid

## Abstract

Carnitine-acylcarnitine translocase deficiency (CACTD) is a rare autosomal recessive fatty acid oxidation disorder resulting in energy deficiency due to impaired mitochondrial long-chain fatty acid transport. Hyperammonemia is a critical complication, often resistant to conventional treatment. Here, we report the case of a 7-month-old patient with CACTD, initially diagnosed at 10 days old, who presented with persistent hyperammonemia despite optimized medical nutrition therapy and conventional nitrogen scavenging with sodium benzoate. When hyperammonemia persisted, carglumic acid was introduced, leading to a sustained decrease in ammonia levels and effective long-term control. Carglumic acid, typically indicated for organic acidemias, proved beneficial in this CACTD case. The administration of carglumic acid not only provided acute resolution but also stabilized ammonia levels over prolonged follow-up. This case highlights carglumic acid as a potential therapeutic option for managing hyperammonemia in CACTD, underscoring the need for further studies to confirm its efficacy in long-term management of hyperammonemia in fatty acid oxidation disorders.

## 1 Introduction

Carnitine- acylcarnitine translocase deficiency (CACTD, MIM: #212138) is an autosomal recessive fatty acid oxidation disorder caused by biallelic pathogenic variants in the gene *SLC25A20* (OMIM: *613698) encoding the enzyme carnitine- acylcarnitine translocase (CACT). Since the mitochondrial CACT enzyme plays an essential role in the transport of long-chain fatty acids across the mitochondrial membrane, where they undergo β-oxidation, reduced enzyme activity leads to energy deficiency, especially during periods of fasting or increased energy requirements [1].

The clinical phenotype of the disease comprises two different forms. The neonatal form is a severe form characterized by poor feeding, hypotonia, lethargy, cardiac arrhythmias, cardiomyopathy, hypoketotic hypoglycemia, hyperammonemia, transaminitis, liver dysfunction with hepatomegaly and rhabdomyolysis. The later-onset form has similar but usually milder symptoms [2,3]. Diagnosis is made by a combination of biochemical tests, including plasma acylcarnitine profiling, which typically shows elevated levels of long-chain acylcarnitines (C16-, C16:1-, C18-, and C18:1- acylcarnitines) and dicarboxylic aciduria in the urine organic acid profile. Definite diagnosis of CACTD is made by the detection of biallelic pathogenic variants in the *SLC25A20* gene [4].

Treatment of CACTD focuses primarily on preventing catabolic states by ensuring adequate energy intake through frequent feeding, a high-carbohydrate, low-fat diet and supplementation with medium-chain triglycerides (MCTs) or triheptanoin, which can bypass the blockade of long-chain fatty acid oxidation [5].

Hyperammonemia is a common and even life-threatening complication of CACTD, as individuals with early-onset CACTD may suffer from recurrent hyperammonemia leading to developmental delay or intellectual disability [6]. Although dietary modification and nitrogen scavenger therapy have been used to treat hyperammonemia in CACTD, there is no consensus [7]. Here we present a 7-month-old patient who developed persistent hyperammonemia despite optimal medical nutrition therapy, that resolved after long-term use of carglumic acid.

## 2 Case report

The patient, who was diagnosed with CACTD at the age of 10 days, was referred to our clinic for further observation at the age of 7 months. The first child of the consanguineous parents died of resistant hipocalcemia and cardiac arrest at the age of three days. For this reason, whole-exome sequencing was performed, which revealed a c.395 A > T (p.Glu132Val) variant in heterozygous state in the *SLC25A20* gene in both parents.

Our patient was born by cesarean section in the 38th week of gestation with a birth weight of 3200 g. She was admitted to the neonatal intensive care unit on the third day of life because she developed severe hypoglycemia and a heart murmur was detected on physical examination. Transthoracic echocardiography revealed arcus hypoplasia, a wide ductus arteriosus, ventricular hypertrophy and hypertrophic cardiomyopathy. Interventricular septal thickness in diastole (IVSD) was measured at 7.1 mm (z-score + 3), and fractional shortening was normal. Her plasma acylcarnitine profile showed decreased free carnitine and increased C16-, and C18- acylcarnitine levels, suggestive of CACTD. Further examination of the *SLC25A20* gene revealed the variant c.395 A > T (p.Glu132Val) in the homozygous state and confirmed the diagnosis. To the best of our knowledge, this variant has not been reported before and is defined as likely pathogenic according to the ACMG criteria in in –silico databases. Medical nutrition therapy with high carbohydrate and low fat with MCT and oral carnitine supplementation was initiated.

At the first examination in our outpatient clinic at the age of 7 months, after referral from another center, the patient presented with mild hypotonia. Physical examination revealed a systolic murmur of 3/6 and hepatomegaly extending 3 cm above the costal margin. Transthoracic echocardiography revealed hypertrophic cardiomyopathy and left ventricular outflow tract obstruction. The IVSD was measured at 9 mm (z-score + 3.79) and fractional shortening was normal. Abdominal ultrasonography revealed slightly increased echogenicity of the liver with a heterogeneous appearance. Plasma creatine kinase (443 U/L), aspartate aminotransferase (100 IU/L) and alanine aminotransferase (108 IU/L) were elevated. The plasma ammonia level was measured at 84 μmol/L. Her plasma acylcarnitine profile on admission showed low levels of free carnitine (2.87 mmol/L) with normal C16-, and C18- acylcarnitine levels. However, the ratio of C16 + C18:1/C2 was 0.29 and C16 + C18/C0 was 1.08. The patient's oral carnitine dose was increased to 100 mg/kg/day.

At routine 9-month follow-up, her plasma ammonia level was elevated to 300 μmol/L, and creatine kinase was measured at 4000 U/L. She had no hypoglycemia, metabolic acidosis, or hyperlactatemia. Because of suspected secondary energy deficiency, her diet was changed to continuous nasogastric tube feeding and energy intake was increased. Despite adequate energy intake and the use of triheptanoin, plasma ammonia levels remained elevated and treatment with sodium benzoate was initiated. As no laboratory improvement in hyperammonemia could be achieved with sodium benzoate, carglumic acid (200 mg/kg/day) was added as a scavenger treatment and plasma ammonia levels decreased to 80 μmol/L. After plasma ammonia levels returned to normal, an attempt was made to discontinue treatment with carglumic acid. However, after discontinuation of treatment, hyperammonemia recurred even though an appropriate diet was maintained. There was no apparent cause for the hyperammonemia, such as infection or noncompliance with medical and nutritional therapy. Treatment with sodium benzoate was discontinued and treatment with carglumic acid was resumed, resulting in a gradual decrease in ammonia levels to 80 μmol/L.

**Fig. 1:**
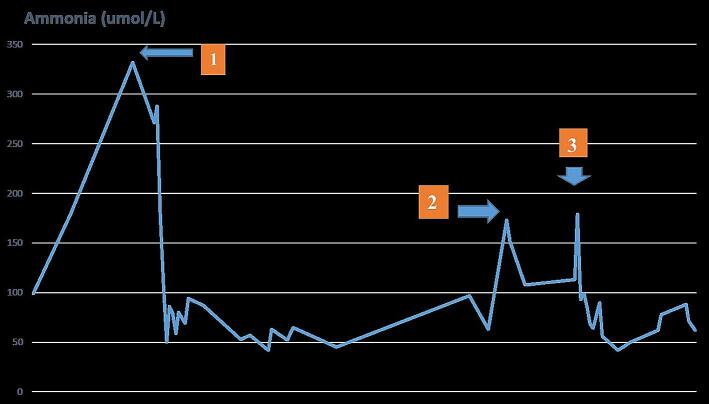
Data on the patient's plasma ammonia levels during the follow-up period: 1. change in medical nutrition therapy, 2. additional treatment with sodium benzoate, 3. addition of carglumic acid treatment.

Subsequently, the medical treatment was supplemented with carglumic acid for 14 months. Dose adjustments were made, with 100–200 mg/kg/day proving effective in controlling the patient's hyperammonemia. During long-term treatment with carglumic acid, the patient's plasma ammonia levels remained within the normal range (Fig. 1). Data on the patient's anthropometric measurements, clinical and laboratory findings from initial referral to 23 months of age are shown in Table 1.

**Table 1:** Data on the patient's anthropometric measurements, medical and nutritional treatments, and metabolic parameters during the follow-up period.

Age	7 months	9 months	12 months	18 months	23 months
Anthropometric measurements					
Height (cm)	71	73	80	86	90
Height z score (SDS)	1.6	1.27	1.72	1.38	1.15
Height-for-age (cm)	66	72	75	82	86
Weight (kg)	8.95	10	12.3	15.8	17
Weight z score (SDS)	1.29	0.67	2.3	3.34	3.12
Weight-for-age (kg)	7.9	8.5	9.4	10.8	11.8
Weight-for-height (percentile)	76.73	92.22	98.38	99.54	99.92
Head circumference (cm)	43	45	46.5	48	49
Head circumference z score (SDS)	0.35	0.3	0.55	0.61	0.8
BMI z score (SDS)	0.55	1.12	1.58	2.87	2.69
Nutrition therapy	116 kcal/kg/d energy, 10 % LCT, 19 % MCT, 11 % prt, 60 % CHO DCI	100 kcal/kg/d energy, 10 % LCT, 20 % Triheptanoin, 10 % prt, 60 % CHO DCI	125 kcal/kg/d energy, 10 % LCT, 30 % Triheptanoin, 9 % prt, 51 % CHO DCI	100 kcal/kg/d energy, 10 % LCT, 20 % Triheptanoin, 10 % prt, 60 % CHO DCI	100 kcal/kg/d energy, 10 % LCT, 35 % Triheptanoin, 8 % prt, 47 % CHO DCI
Longest permitted fasting time	2 h	Continuous 24 h feeding	Continuous 24 h feeding	Continuous 24 h feeding	Continuous 24 h feeding
Medical treatment for hyperammonemia		-Sodium benzoate (250 mg/kg/day) -Carglumic acid (200 mg/kg/day)	-Sodium benzoate (250 mg/kg/day) -Carglumic acid (200 mg/kg/day)	-Carglumic acid (100 mg/kg/day)	-Carglumic acid (150 mg/kg/day)
Laboratory findings					
Ammonia (μmol/l)	84	332	173	56	90
CK (U/L)	443	249	268	4313	1022
AST (IU/L)	100	102	67	488	150
ALT (IU/L)	108	41	29	205	51
Plasma acylcarnitine profile					
C16	2.41	3.42	3.5	3.5	6.35
C18	0.69	1.33	0.71	1.41	1.8
C18:1	0.92	2.53	2.13	1.48	2.75
C18:2	0.06	0.38	0.14	0.28	0.29
C16 + C18/C0	1.08	0.33	3.6	0.5	0.94
C16 + C18:1/C2	0.29	0.39	1.33	0.16	0.6

SDS, standard deviation score; BMI, body mass index;CK, creatine kinase; AST, aspartate aminotransferase; ALT, alanine aminotransferase;prt, protein; CHO, carbonhydrate; LCT, long-chain triglycerides; DCI, daily caloric intake; MCT: medium-chain triglycerides.

### 2.1 Management of medical nutrition therapy

The medical nutrition therapy that the patient was receiving at the time of her initial admission to our outpatient clinic was 80 kcal/kg/d of energy, 18 % below the RDA recommendations [8]. The macronutrient distribution was 10 % long-chain triglycerides (LCT), 15 % medium-chain triglycerides (MCT), 60 % carbohydrates (CHO) and 15 % protein (prt) of the daily caloric intake (DCI). The patient was fed orally, with the longest permitted fasting time being 2 h.

At the patient's 9th month routine checkup, hyperammonemia was detected, the energy content of the diet was increased and MCT was changed to triheptanoin. Her medical nutrition therapy consisted of 100 kcal/kg/d, 10 % LCT, 20 % triheptanoin, 60 % CHO, 10 % prt DCI. During hyperammonemia, she was continuously fed via a nasogastric tube for 24 h. Subsequently, the patient's triheptanoin intake was gradually titrated to 35 % of the DCI values, as recommended in the literature [9].

During the follow-up period, regular food intake records and telephone interviews demonstrated that the patient fully adhered with the prescribed medical nutrition therapy. Data on the details of medical nutrition therapy and plasma ammonia levels are shown in Table 1.

## 3 Discussion

In this report, we presented a case of CACTD characterized by persistent hyperammonemia. Despite optimal nutritional management, and no other apparent cause, the patient's ammonia levels remained elevated. The patient did not respond adequately to conventional ammonia scavenger, sodium benzoate. However, the administration of carglumic acid proved successful in controlling the hyperammonemia.

Hyperammonemia is a frequent and challenging complication of CACTD, occurring in over 50 % of cases. The exact mechanism underlying hyperammonemia in CACTD is not fully understood, and there is limited real-time data during acute decompensation. Secondary hyperammonemia may arise from impaired ureagenesis due to the accumulation of toxic metabolites or deficiencies in key substrates required for the urea cycle. One proposed mechanism suggests that insufficient mitochondrial acetyl-CoA disrupts *N*-acetylglutamate synthesis, leading to decreased carbamoyl phosphate synthetase 1 (CPS1) activity and impaired urea cycle function [10,11].

Studies have demonstrated that high rates of intravenous dextrose represent the most effective strategy for the acute treatment of hyperammonemia in CACTD. However, there are several treatment approaches for the chronic management of hyperammonemia, including modifications of nutritional therapy and medical treatments, all of which have limited clinical efficacy. A high dietary carbohydrate intake, although effective, can lead to excessive weight gain, hepatomegaly, and steatohepatitis in the long term. Interestingly, higher protein intake (2–4 g/kg/day) has been associated with greater metabolic stability and improved hyperammonemia, possibly due to the induction of urea cycle enzymes or an anaplerotic effect. This dietary adjustment has also been associated with a reduction in hospitalizations and a lower incidence of complications related to steatosis, compared to a high intake of medium-chain triglycerides (MCTs) or carbohydrates [7,12,13]. Apart from the glucose response, hyperammonemia in this cohort appears to respond favorably to sodium D,L-3-hydroxybutyrate and increased dietary protein intake, both of which induce anaplerosis [7,14]. Nitrogen-scavenging drugs, such as sodium benzoate and sodium phenylbutyrate, generally show limited efficacy in these cases [7,15].

Carglumic acid is an orphan drug and a derivative of *N*-acetylglutamate, which activates the first enzyme—CPS1—in the urea cycle. It is primarily indicated for the treatment of hyperammonemia associated with *N*-acetylglutamate synthase deficiency, carbonic anhydrase Va deficiency, and some organic acidemias. Regarding hyperammonemia associated with mitochondrial fatty acid oxidation disorders, its efficacy has been documented in multiple acyl-CoA dehydrogenase deficiency [16]. Data on the use of carglumic acid in CACTD were reported in a case demonstrating the beneficial effects of carglumic acid in combination with sodium phenylbutyrate in the treatment of acute hyperammonemia [17].

In our case, hyperammonemia was initially managed through dietary measures, such as increasing caloric intake, the use of triheptanoin, and continuous feeding via a nasogastric tube. However, as ammonia levels remained elevated, nitrogen scavenger treatment was administered. The hyperammonemia was eventually successfully treated with carglumic acid. Treatment with carglumic acid proved to be an effective strategy not only in the acute phase but also in the long-term management of the patient's hyperammonemia.

## 4 Conclusion

The treatment of CACTD requires a comprehensive strategy that includes close monitoring of metabolic parameters, personalized nutritional interventions and careful use of pharmacological agents to reduce the risk of hyperammonemia. While triheptanoin remains the first-line option in the nutritional treatment of CACTD, its efficacy may be limited in certain cases. In such situations, carglumic acid should be considered as adjunctive therapy alongside triheptanoin, particularly for the treatment of persistent hyperammonemia. The positive outcome observed in this case suggests that carglumic acid could be a valuable option of CACTD-related hyperammonemia, but further studies are needed to clarify its long-term efficacy and optimal use.

## Author contribution

HB, TZ, and CAZ conceptualized and designed the study, drafted the initial manuscript, and critically reviewed and revised the manuscript.

HB, EU, MSC and EK designed the data collection instruments, collected data, carried out the initial analyses, and critically reviewed and revised the manuscript.

conceptualized and designed the study, coordinated, and supervised data collection, and critically reviewed and revised the manuscript for important intellectual content.

All authors approved the final manuscript as submitted and agree to be accountable for all aspects of the work.

Tanyel ZUBARIOGLU is the corresponding author of this study.

## Funding/support

There is no funding available.

## Ethical approval

Ethics approval was not required because it is a retrospective case report.

All procedures followed were in accordance with the ethical standards of the responsible committee on human experimentation (institutional and national) and with the Helsinki Declaration of 1975, as revised in 2000.

## Informed consent

Informed consent was obtained from patient for being included in the study.

## CRediT authorship contribution statement

**Hanım Babazade:** Writing – original draft, Methodology, Formal analysis, Data curation, Conceptualization. **Tanyel Zubarioglu:** Writing – review & editing, Writing – original draft, Methodology, Data curation, Conceptualization. **Esma Uygur:** Writing – review & editing, Methodology, Formal analysis. **Mehmet Şerif Cansever:** Methodology, Formal analysis. **Ertuğrul Kiykim:** Writing – review & editing, Writing – original draft, Formal analysis, Data curation, Conceptualization. **Çiğdem Aktuğlu Zeybek:** Writing – review & editing, Writing – original draft, Methodology, Formal analysis, Data curation, Conceptualization.

## Declaration of competing interest

Authors state no conflict of interest.

## Data Availability

The data that support the findings of this report are available from the corresponding author upon reasonable request.
